# DEVELOPING A TAILORED STRAIN INDEX FOR LONG-DISTANCE FAMILY CAREGIVERS OF PERSONS WITH DEMENTIA

**DOI:** 10.1093/geroni/igae098.3376

**Published:** 2024-12-31

**Authors:** Francesca Falzarano, Verena Cimarolli, Kathrin Boerner, Richard Chunga

**Affiliations:** University of Southern California, Los Angeles, California, United States; LeadingAge, Washington, District of Columbia, United States; Carl von Ossietzky University of Oldenburg, Oldenburg, Niedersachsen, Germany; University of Massachusetts Boston, Boston, Massachusetts, United States

## Abstract

Most research has examined the experiences and consequences of dementia family caregiving among proximate caregivers, while the unique needs of the growing number of long-distance caregivers (LDCs) remain understudied. Since the vast majority of research and evidence-based intervention efforts have specifically focused on caregivers who reside in close proximity to their care-recipients, existing measures of caregiver strain do not adequately capture the unique distance-related challenges experienced by LDCs. Thus, the current study aims to evaluate the suitability of a nine-item strain index tailored for LDCs. The scale is comprised of: 1) a three-item measure assessing level of difficulty experienced with the physical, financial, and emotional aspects of care from the National Study on Caregiving (NSOC); and 2) six novel items assessing difficulty with LDC (e.g., monitoring care, traveling to the care-recipient). Baseline data (n=40) derived from a study examining the feasibility of LDCare - a virtual, multicomponent intervention for LDCs - were used to conduct an exploratory factor analysis (EFA) with the NSOC and LDC-specific items. Initial findings suggested the exclusion of two factors that did not significantly contribute to the scale’s underlying covariance structure. Further analyses suggested that a seven-item scale comprised of two components yielded the best model fit, accounting for 57.1% of the total variance. The final scale included three items capturing distance challenges and four items assessing general caregiving challenges. Findings provide evidence for the scale’s suitability among LDCs and underscore the importance of tailored assessments to adequately measure the unique challenges associated with LDC.

